# Design and Test of a Calibration System for Avalanche Photodiodes Used in X-Ray Compton Polarimeters for Space

**DOI:** 10.3390/s24248016

**Published:** 2024-12-15

**Authors:** Andrea Alimenti, Fabrizio Cologgi, Sergio Fabiani, Kostiantyn Torokhtii, Enrico Silva, Ettore Del Monte, Ilaria Baffo, Sergio Bonomo, Daniele Brienza, Riccardo Campana, Mauro Centrone, Giulia De Iulis, Enrico Costa, Giovanni Cucinella, Andrea Curatolo, Nicolas De Angelis, Giovanni De Cesare, Andrea Del Re, Sergio Di Cosimo, Simone Di Filippo, Alessandro Di Marco, Giuseppe Di Persio, Immacolata Donnarumma, Pierluigi Fanelli, Abhay Kumar, Paolo Leonetti, Alfredo Locarini, Pasqualino Loffredo, Giovanni Lombardi, Gabriele Minervini, Dario Modenini, Fabio Muleri, Silvia Natalucci, Andrea Negri, Massimo Perelli, Monia Rossi, Alda Rubini, Emanuele Scalise, Paolo Soffitta, Andrea Terracciano, Paolo Tortora, Emanuele Zaccagnino, Alessandro Zambardi

**Affiliations:** 1 Department of Industrial, Electronic and Mechanical Engineering, Roma Tre University, Via V. Volterra 62, 00146 Rome, Italy; fab.cologgi1@stud.uniroma3.it (F.C.); kostiantyn.torokhtii@uniroma3.it (K.T.); 2 INAF-IAPS, Via del Fosso del Cavaliere 100, 00133 Rome, Italynicolas.deangelis@inaf.it (N.D.A.);; 3 Dipartimento di Economia, Ingegneria, Società e Impresa, Università degli Studi della Tuscia, Largo dell’Università, 01100 Viterbo, Italy; 4 IMT s.r.l., Via Carlo Bartolomeo Piazza 30, 00161 Rome, Italy; 5 Agenzia Spaziale Italiana, Via del Politecnico snc, 00133 Rome, Italy; 6 INAF-OAS Bologna, Via Piero Gobetti 93/3, 40129 Bologna, Italy; 7 INAF-OAR, Via Frascati 33, 00040 Monte Porzio Catone, Italy; 8 SCAI Connect s.r.l., Via Vincenzo Lamaro 51, 00173 Rome, Italy; 9 Department of Industrial Engineering, Alma Mater Studiorum Università di Bologna, Via Fontanelle 40, 47121 Forlì, Italydario.modenini@unibo.it (D.M.);; 10 Dipartimento di Ingegneria dell’Impresa “Mario Lucenti”, Università degli Studi di Roma “Tor Vergata”, Via Cracovia 50, 00133 Rome, Italy; 11 INAF Headquarters, Viale del Parco Mellini 84, 00136 Rome, Italy; 12 Interdepartmental Center for Industrial Aerospace Research, Alma Mater Studiorum Università di Bologna, Via Fontanelle 40, 47121 Forlì, Italy

**Keywords:** X-ray polarimeter, avalanche photodiode, measurement system for APD calibration

## Abstract

The development and calibration of a measurement system designed for assessing the performance of the avalanche photodiodes (APDs) used in the Compton scattering polarimeter of the CUSP project is discussed in this work. The designed system is able to characterize the APD gain $G_{APD}$ and energy resolution across a wide range of temperatures *T* (from −20 °C to +60 °C) and bias voltages $V_{bias}$ (from 260 V to 410 V). The primary goal was to experimentally determine the $G_{APD}$ dependence on the *T* and $V_{bias}$ in order to establish a strategy for stabilizing $G_{APD}$ by compensating for *T* fluctuations, acting on $V_{bias}$. The results demonstrate the system capability to accurately characterize APD behavior and develop feedback mechanisms to ensure its stable operation. This work provides a robust framework for calibrating APDs for space environments. It is essential for the successful implementation of spaceborne polarimeters such as the Compton scattering polarimeter foreseen aboard the CUbeSat Solar Polarimeter (CUSP) mission under development to perform solar flare X-ray polarimetry.

## 1. Introduction

Solar flares are sudden and intense releases of energy originating in the Sun’s Corona, caused by a rearrangement of the magnetic field lines via magnetic reconnection. These powerful eruptive events emit a broad spectrum of electromagnetic waves, including X-rays and ultraviolet light [1]. Along with electromagnetic radiation, solar flares also accelerate charged particles, such as protons and electrons, which can travel through space and reach the Earth’s magnetosphere. Moreover, solar flares are also very often associated with the release of huge structures of magnetic field trapping charged particles that travel in interplanetary space and are known as coronal mass ejection (CME) [1,2,3,4].

The impact of solar flares on human technology is significant [5,6,7]. While the Earth’s magnetic field and atmosphere allow shielding from a lot of the harmful charged particles and electromagnetic radiation, the high-energy components can still cause relevant geomegnetic storms that disrupt satellite communications, interfere with power grids, and cause inaccuracies in GPS systems. The release of energy in the Earth ’s atmosphere can also induce its expansion, increasing the drag on satellites and leading to potential operational challenges and reduced lifespans for these spaceborne assets. Additionally, solar flares can pose a radiation hazard to astronauts in space and passengers on high-altitude flights near the poles.

Understanding and predicting solar flares is crucial for mitigating their effects on our technological infrastructure [8]. By monitoring solar activity and studying the mechanisms behind these powerful phenomena, scientists aim to develop better predictive models and protective measures [9]. This proactive approach helps to safeguard critical systems, ensuring the reliability and resilience of our technology-dependent society. In particular, X-ray polarimeters are valuable instruments capable of determining the properties of the magnetic fields originating the solar eruptions and measuring also the directivity [10] and beaming properties of accelerated particles. The anisotropic distributions of electrons accelerated in ordered magnetic fields produce non-thermal emissions above 10–20 keV that are expected to be significantly polarized on the basis of various models [10,11,12,13,14,15].

To this end, the CubeSat Solar Polarimeter (CUSP) project [16] aims to develop a constellation of two CubeSat satellites orbiting the Earth to significantly contribute to the current and future network of infrastructures useful for Space Weather monitoring activities such as the ASPIS (ASI SPace Weather InfraStructure) project of the Italian Space Agency (ASI) [17]. CUSP is a project funded by the Alcor program of ASI. Specifically, it will utilize Compton scattering polarimeters to measure the linear polarization of solar flares in the hard X-ray band (25–100 keV). The objective is to develop compact polarimeters, capable of flying on CubeSats, without optics, and achieving a minimum detectable polarization (MDP [18,19]) of 10.2% for an M5.2 class flare [16].

To achieve this, the CUSP payload will consist of a dual-phase Compton scattering polarimeter to perform coincidence measurements between signals from plastic and inorganic scintillators. Specifically, the light emitted by the plastic scintillators will be read out by multianode photomultipliers (MAPMTs), and the scattered photons will be absorbed by Gd_3_Al_2_Ga_3_O_12_ (GAGG) inorganic scintillators, with the resulting light signal read out by an array of 32 avalanche photodiodes (APDs). GAGG scintillators are chosen to maximize the photon absorption probability, thanks to their high effective atomic number [16], while platic scintillators are chosen to maximize scattering probability.

Due to the very short implementation time required for this mission, well-known components, already used for this kind of application, have been selected. This reduces the risk of potential issues during the implementation, ensuring a higher likelihood of mission success. So, the chosen MAPMTs are the Hamamatsu R7600-03-M16 (rugged version) and the APDs are an SMD version of the Hamamatsu S8664-55 (the same aboard the TSUBAME satellite, unfortunately lost in 2015 [20]).

A precise calibration of each component of the system is essential to achieve the desired metrological characteristics of the polarimeter. To this end, this work addresses the issue of calibrating the response of the APDs under flight operational conditions [21]. Specifically, it is necessary to characterize the gain $G_{APD}$ and energy resolution δE/E of these components as a function of temperature (from −20 °C to +60 °C—typical temperatures that include the range variation of the payload of Earth-orbiting CubeSats [22,23]) and bias voltage $V_{bias}$ (from 260 V to 410 V—typical bias voltage used on APDs; the lower limit corresponds to a low gain value, and the upper value is limited by the breakdown voltage in the device [24,25]). Once the experimental dependence of the gain on temperature $G_{APD}\left(T\right)$ is determined, given the temperature variability to which CubeSat payloads are subjected, a feedback system will be developed to maintain the APD gain stable despite *T* fluctuations by adjusting $V_{bias}$. This work thus analyzes the challenge of calibrating APDs over such a broad parameter space, develops an appropriate measurement system, and preliminarily analyzes the data to establish possible control curves for stabilizing the gain of the APDs.

This paper is organized as follows. In Section 2, a brief introduction to the APD is provided to the reader, focusing on the parameters (i.e., $G_{APD}$ and δE/E) that will be characterized in this work, highlighting the expected dependencies of these to *T* and $V_{bias}$. Then, in Section 3, the design of the measurement system is discussed in detail. In particular, first, the electronic circuits and measurement instrumentation for the characterization of $G_{APD}$ and δE/E are presented, and then the system for the control of the temperature and humidity, which is based on a climate chamber, is introduced. Once the developed system is described, the measurement procedure and the system calibration and setup are discussed in Section 4. Due to the numerous components that elaborate the analog signal from the APD to the digitization stage, it becomes difficult, and it would not guarantee particularly accurate results, to attempt to calibrate the losses and gains introduced by each component in the line to assess the response of the entire system. To address this, it will be shown how a previously calibrated APD was used as a reference to calibrate the entire system. Finally, in Section 5, the measurements obtained are analyzed in detail, and conclusions are provided in Section 6.

## 2. Avalance Photodiodes (APDs)

The performance of the X-ray polarimeter strongly depends on the performance of the photodetectors used. Thus, the characterization of photodetectors is fundamental for the right operativity of the final device. The measurement of the detector parameters is an interesting challenge from the instrumentation and measurement point of view since it involves the measurement of a large number of different physical quantities that must be accurately controlled in the space parameters of interest for the operative scenario.

This section provides a brief introduction to avalanche photodiodes (APDs), focusing on the physical quantities that this work aims to characterize.

APDs are semiconductor devices used in the detection of low-level radiation signals. Their principle of operation is the same as standard photodiodes: when a photon with energy higher than the energy band gap arrives in the sensitive region, an electron–hole pair can be generated. The ratio between the number of generated pairs to the number of impinging photons is called the quantum efficiency (QE) of the device [24,26].

Apart from the photocurrent-generating mechanism, APDs differ from photodiodes due to their ability to multiply the photocurrent. The high electric field applied to the PN junction accelerates the carriers to a drift speed that is useful for ionizing the atoms in the crystal lattice during the collisions. This creates a chain reaction called avalanche multiplication. The entity of this multiplicative effect is described by the APD gain $G_{APD}$ [25,26,27]. As long as the APD leakage current can be neglected, $G_{APD}$ can be defined as:

(1) $$G_{APD}\approx I_{D}/I_{dg}\;,$$

where $I_{D}$ is the total APD dark current and $I_{dg}$ the dark current generated inside the APD substrate.

The multiplicative effect, i.e., $G_{APD}$, depends on the drift velocity of the carriers. Thus, $G_{APD}$ is expected to be dependent on the externally applied voltage $V_{bias}$. In addition to this, the temperature *T* also plays an important role: as the temperature increases the lattice vibration of the APD semiconducting material, the possibility of collision between the accelerated carriers and the lattice itself also increases, even before the carriers reach a sufficiently high speed to generate ionization, thus bringing a reduction in $G_{APD}$ [25,28]. One of the most used models describes all these phenomena with the following exponential law [29]:

(2) $$G_{APD}\left(T,V_{bias}\right)=a+be^{\left(cV_{bias}+dT\right)}\;,$$

where *a*, *b*, *c*, and *d* are device-dependent parameters. Other models [29,30] are also used in the literature to describe the dependence of $G_{APD}$ on *T* and $V_{bias}$ in restricted parameter spaces. The different models are detailed and further discussed in Section 5.

It is worth noting that the so-defined $G_{APD}$ is a measure of the multiplication of charge carriers, and thus it does not include the QE of the device. This means that $G_{APD}$ does not depend on the wavelength of the incident photons or other extrinsic phenomena.

Due to the $G_{APD}$ dependence on *T*, applications involving APDs in environments characterized by *T* variations impose the temperature characterization of their $G_{APD}$. In this work, a measurement system able to measure $G_{APD}$ over the temperature range expected in orbiting CubeSats is designed. In particular, the bi-dimensional function given by (2) will be experimentally determined in order to exploit the possibility of controlling $V_{bias}$ to compensate the effects of *T* variations on $G_{APD}$.

Another important physical property of APDs that can affect the final performance of a polarimeter is energy resolution [25,31]. This refers to the ability of the device to distinguish between photons of different energies. It is quantified by the ratio δE/E, where δE represents the full width at half maximum (FWHM) of the peak corresponding to a particular energy in the energy spectrum, and *E* is the centroid (mean) of that peak. A smaller δE/E indicates better energy resolution, meaning the detector can more precisely differentiate between energies. This metric is crucial in fields such as spectroscopy, where precise energy measurement is essential.

In APDs, δE/E depends on factors such as the gain of the device, the noise characteristics, and the intrinsic properties of the semiconductor material [25]. Higher $V_{bias}$ increases the gain, improving the signal-to-noise ratio, but it also increases noise, which can degrade resolution. Thus, a minimum is expected in δE/E when $V_{bias}$ is changed. Temperature *T* also has a significant impact on energy resolution; as temperature increases, thermal noise increases, leading to poorer resolution. Conversely, cooling the APD can enhance resolution by reducing noise.

Therefore, optimal δE/E is achieved by balancing the $G_{APD}$ and noise through careful control of *T* and $V_{bias}$.

## 3. Design of the Measurement System

In this section, the developed measurement system is described.

**Figure 1:**
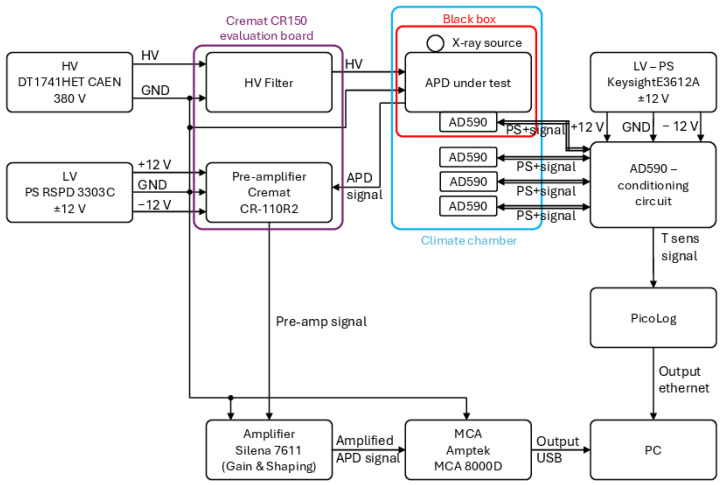
Block diagram of the designed and tested measurement system.

The system block diagram is shown in Figure 1. It is composed of a part that aims at the measurement of $G_{APD}$ and δE/E and a second part useful for controlling and monitoring the temperature in the climate chamber. The two parts are described in the following subsections.

### 3.1. Design of the System for Gain and Energy Resolution Measurement

In order to measure the APD $G_{APD}$ and δE/E, it is necessary to have a photon source, a high voltage generator to properly bias the APD, electronic circuits for the APD output signal conditioning, and a measurement instrument for the data readout [21]. These different parts are here described in detail:

- *Photon generators*: In order to measure the quantities of interest, the APD under test must be properly excited. In this work, we used the following radioisotopes as X-ray photon sources: ^55^Fe, ^109^Cd, ^241^Am, and ^57^Co. Details on the sources are given in Table 1 [32].

**Table 1:** List of the radioisotopes used as X-ray sources [33].

Radioactive Isotope	Half-Life (y)	Main (Effective) Emitted Line (keV)
^55^Fe	2.737	5.9
^109^Cd	1.263	22.6
^241^Am	432.6	59.5
^57^Co	0.745	123.6
- *APD biasing system*. As discussed in Section 2, APDs are designed to work with a large inverse polarization voltage. Thus, a high-voltage (HV) source is needed. In this work, the HV is provided by the DT1471HET CAEN HV generator. It is a four-channel HV power supply with 5.5 kV maximum output, with an accuracy of ±2% of the read value ±2 V and 20 mVpp maximum voltage ripple. In order to reduce the voltage instabilities, the supply voltage filter of the CR-150 evaluation board is used. It is a dual-pole RC filter composed of a first 100 MΩ with 0.01 μF low-pass RC filter followed by a second filter realized with a 200 MΩ resistor and 0.01 μF capacitor. The poles of this filter can be changed by shunting the resistors depending on the dark current of the connected APD. The dark current of the APD under test is nominally 5 nA at 25 °C, and thus no shunt resistors are added to the filter accordingly with the datasheet of the CR-150 evaluation board [34].
- *APD signal output conditioning system*. For each electron that is possibly generated by the absorption of an incident photon in the APD sensitive region, a charge pulse is generated by the avalanche phenomenon. The measurement of the quantity of charge contained in the pulse would allow us to directly determine $G_{APD}$. A possible alternative would consist of transforming the physical quantity that carries the information into another quantity more easily measured: for example, the voltage. In this work, the second possibility is chosen due to the more common, and generally available, laboratory instrumentation measurement. Thus, the charge pulses are passed to the charge preamplifier which is mounted on the CR-150 evaluation board [34]. The CR-110R2.2 is a charge-sensitive preamplifier widely used with several kinds of radiation detectors [35]. The equivalent circuit of the CR-110R2.2 is shown in Figure 2. It is a dual-stage amplifier: the first one is a charge preamplifier followed by a low-gain voltage amplifier. Thus, each charge pulse is integrated by the $C_{f}\approx 1.4$ pF capacitor. Since the preamplifier output voltage is $v_{out}=q/C_{f}$, with *q* the quantity of charge, then the preamplification conversion factor is $V_{out}/q=1/C_{f}=0.7$ V/pC.
  The feedback resistor $R_{f}$ slowly discharges the capacitor, giving the exponential decay of the output voltage. The characteristic time is $\tau =R_{f}C_{f}\approx 140\;\mu$s. Thus, the detector output charge pulses should be limited in duration to a few microseconds, since for longer pulses the exponential decay would distort the shape and carried information. The pulse rise time of this preamplifier is 7 ns and the overall gain of the dual-stage preamplifier is ≈1.4 V/pC.
  Due to the random (Poisson) statistics of the APD emitted charge pulses (once one of the X-ray sources is placed in contact), it is not possible to exclude the possibility that two pulses are emitted so close that the $C_{f}$ is not completely uncharged before the arrival of the second pulse. This would bring a pile-up of the pulses, providing incorrect amplitude information. To avoid this phenomenon, pulse shaping stages are usually used. The pulse-shaping stage aims to provide an output of well-separated pulses, avoiding their overlap, and without losing/distorting the information of the preamplifier signal [35]: pulse amplitude and time. The easiest way to accomplish this is by using high-pass RC filters and differentiation circuits; the output of these circuits enhances the fast variations of the voltage v(t) signal corresponding to the arrival of a new photon, reducing the effect, if properly designed, of the long tails of the preamplifier output signal. Alternatively, Gaussian filters are more commonly used. These are filters whose impulse response is an approximation of the Gaussian function. This kind of filter is generally integrated with spectroscopy amplifiers. In this case, the Silena 7611 amplifier is used. Its pulse-shaping stage is based on a Gaussian filter with variable shaping time $0.25<t_{s}/\left(\mu s\right)<6$, and it is followed by a voltage amplifier whose gain can be continuously regulated by the user from 2.5 to 3000. The set-up of the system will require a fine-tuning of these parameters, i.e., $t_{s}$ and the gain of the Silena 7611 amplifier.
  The signal elaboration chain from the APD to the amplifier output is summarized in Figure 3.
- *Readout system*. Once the signal is properly conditioned, it can be measured. Since the information about the quantity of charge generated is preserved in the amplitude of the voltage pulse output from the spectroscopy amplifier, a system capable of classifying voltage pulses based on their amplitude is required. A multi-channel analyzer (MCA), operating in pulse height analysis (PHA) mode, can be used for this aim. MCAs are based on fast ADC used to digitize the incoming pulses and collect information about these. In the PHA mode, pulses are classified based on their peak voltage. Thus, the output is a histogram where each class (channel) contains the number of pulses counted during the measurement time with an amplitude corresponding to the channel number. Thus, the MCA resolution depends on the number of channels in this operation mode.
  Since scintillators in spectroscopy experiments are often used to convert the energy of the incident in a proportional quantity of charge, photons with different energies correspond to voltage pulses with different amplitudes. Thus, once the MCA channels are calibrated in energy, then the output of the MCA, after a certain measurement time, directly reports the spectra of the radiation incident on the detector. In this work, we will mainly exploit the MCA channel not to discriminate photons with a different energy but to evaluate how $G_{APD}$ changes when different $V_{bias}$ are applied at different *T*. In this work, the Amptek MCA8000D is used.

### 3.2. Climate Chamber System

In order to perform the temperature calibration of $G_{APD}$, measurements are performed in the Angelantoni-DY2000 climate chamber. Before the calibration procedure, additional temperature sensors are placed in the chamber to monitor temperature homogeneity, and special attention is given to humidity control to avoid water condensation on the APDs. In this section, the network of monitoring temperature sensors and the humidity control system are described.

#### 3.2.1. Temperature Monitoring

Due to the long wires needed to connect the measurement instrumentation with the temperature sensors that are opportunely placed in the climate chamber, temperature-to-voltage transducers are to be avoided because of possible voltage drops along the wires. Thus, in this case, AD590 linear temperature transducers were used [36]. They produce an output current proportional to the absolute temperature, with a sensitivity of 1μA/K, in a wide temperature range of $-55<T/{(}^{\circ }C)<+150$. The AD590 is calibrated in order to provide an output current $I_{T}=298.2\;\mu A$ at 298.2 K (25 °C).

Since commonly used DAQ or data loggers usually provide only the possibility of measuring voltage signals, the output of the AD590 must be properly converted into a voltage signal if one wants to exploit this measurement instrumentation. In particular, in this work, the Pico Data Logger was used to monitor the whole network of sensors.

**Figure 4:**
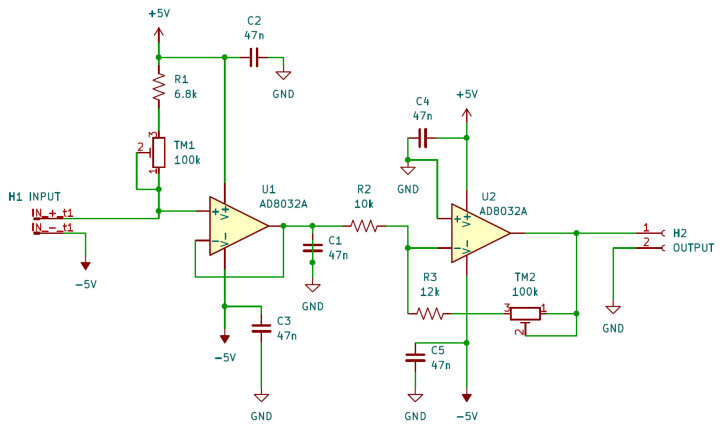
Wire diagram of the electronic circuit realized to adapt the current output of the AD590 temperature transducers to the input characteristics of the PicoLog.

The circuit used for this aim is shown in Figure 4. It aims not only to convert the temperature information to a voltage signal but also to adapt this signal to the analog input characteristics of the PicoLog. Thus, the linear current output of the AD590 must be transformed through a linear current-to-voltage converter that allows the setting of the offset and gain. Thus, the $TM_{1}$ trimmer, which is mounted in series to the $R_{1}$ resistance, regulates the voltage offset. Then, the signal is passed to the voltage buffer realized with the AD8032A amplifier. Finally, the signal is amplified by the following stage, and the output voltage is:

(3) $$V_{out}=-\left(5\;V-\left(R_{1}+R_{TM1}\right)I_{out}\right)\frac{R_{3}+R_{TM2}}{R_{2}}$$

In order to properly regulate the $TM_{1}$ and $TM_{2}$ trimmers, all the AD590 sensors are placed in thermal contact with a metal aluminum bar and thermalized in the climate chamber. The $TM_{1}$ and $TM_{2}$ regulation aims to best exploit the Picolog input dynamic range. So, the $V_{out}=0$ V is set for the lowest needed temperature, −30 °C, acting on the resistance of the TM1 trimmer, and $V_{out}=2.5$ V (the maximum input voltage of the PicoLog) at the maximum temperature of interest, $T_{max}=70$ °C.

The so-realized system was used to monitor temperature drifts on the APD during the measurements.

#### 3.2.2. Humidity Control

**Figure 5:**
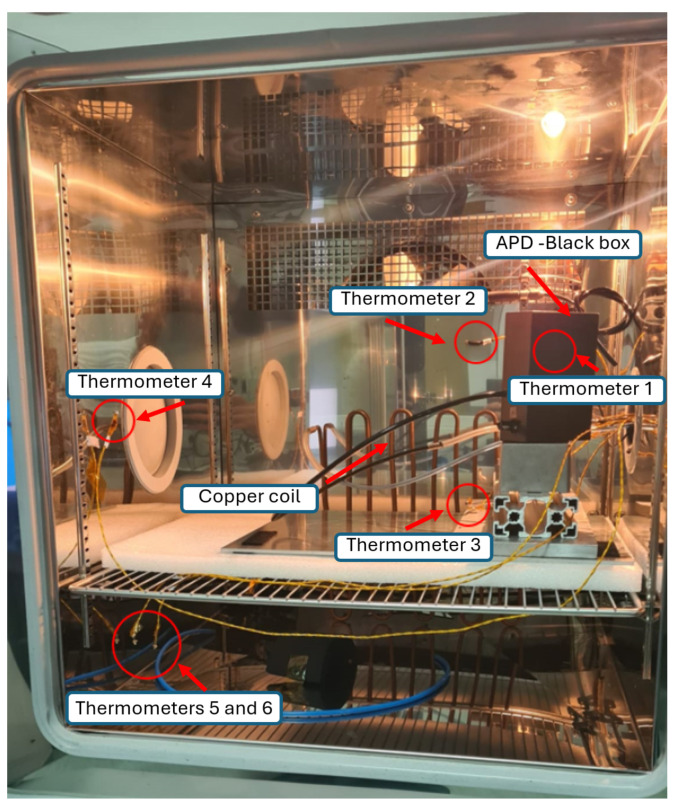
Picture of the measurement setup loaded into the climate chamber.

In order to avoid the condensation of water on the electronic devices loaded into the climate chamber shown in Figure 5, the humidity was carefully monitored and controlled. The chamber used allows the fine control of the relative humidity from 10% to 95%, within ±3%, when the temperature T>5 °C. Since it is necessary to take measurements at lower temperatures as well, particular attention has been paid to the atmosphere introduced into the climate chamber. Specifically, nitrogen gas was flowed into the metal box, suitably darkened, inside which the APD under test was placed. However, the nitrogen gas was passed through a long copper coil inside the chamber to avoid introducing gas at a different temperature than the set one. The temperature transducer mounted near the APD was therefore essential for monitoring any temperature variations caused by the nitrogen flow. Meanwhile, the entire climate chamber was connected to a dry-air generator.

## 4. System Set-Up and Measurement Procedure

**Figure 6:**
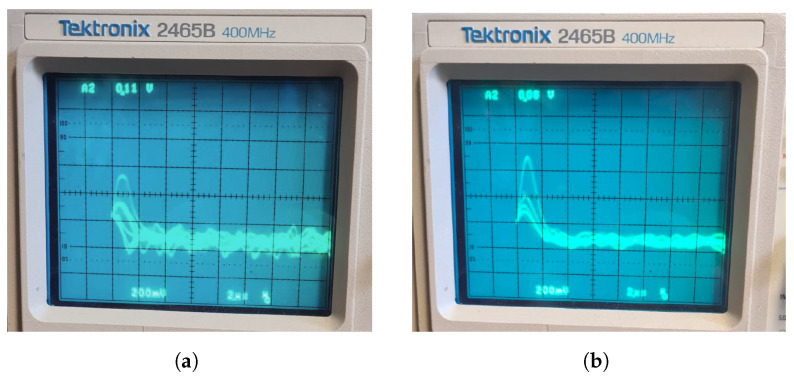
Effect of different wiring in the system on the voltage pulses generated by the spectroscopy amplifier. (**a**) The pulses when a 150 cm long LEMO cable is used. (**b**) When the cable used in (**a**) is substituted with one 15 cm long. In both pictures, the vertical scale is set to 200 mV/div.

Before starting to use the system for the APD calibration, an accurate tuning of the parameters acting on it and its calibration are necessary. To accomplish this, a reference APD can be used. In fact, the APD gain measurement would be possible with the system described here only if all the parts of the system itself, including cables and connectors, were properly calibrated. In order to visualize the effect of different cables on the signal sent as input to the MCA, in Figure 6 the shaped pulses, visualized on an analog oscilloscope, are shown when LEMO cables of 150 cm or 15 cm are used to connect the APD to the preamplifier. Apart from the increased noise level, the peak heights are also different, highlighting the attenuation effect of the cables. Since the quantitative evaluation of the losses introduced by each part of the system would not only be tricky but also unlikely to yield very accurate results, in this work, a pre-calibrated APD is used as a reference to calibrate the entire system.

In this work, the Si S8664-55 Hamamatsu photodiode, serial number AA4400, was used as a reference. For this diode, the calibration certificate states that its breakdown voltage is $V_{b}\approx 433$ V at 25 °C, and, at the same temperature, with $V_{bias}=\left(382.3\pm 0.1\right)$ V, the gain $G_{APD,ref}=50\pm 1$. The number following the ± symbol is to be read as the numerical value of an expanded uncertainty with 100% probability level based on a uniform distribution. This APD will be used to calibrate the whole measurement system.

The two main parameters of the system that can affect its metrological performance are the amplification gain and shaping time. So, by placing a ^55^Fe sample on the APD, the shaped pulses were collected by the MCA and the amplification gain was set to exploit the MCA dynamic range properly, and the shaping time $t_{s}$ was set at a value sufficently low so that the effect of this parameter on the measured energy resolution was negligible; see also below. To this aim, the Silena 7611 gain is set to 210 and several peaks are measured for different $t_{s}$ values to evaluate its effect.

**Figure 7:**
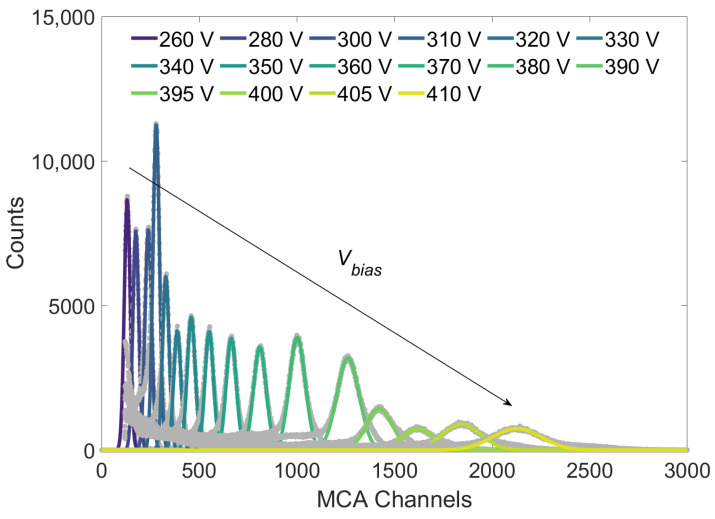
Histograms of the heights of the voltage peaks in input to the MCA and for different $V_{bias}$ values. The grey points are the experimental data and the continuous lines are the fitted Gaussian curves. Data are obtained by placing the ^55^Fe sample directly in contact with the APD, at room temperature. The arrow indicates the direction in which the centroid shifts when $V_{bias}$ is increased.

Figure 7 shows an example of the histograms formed by the MCA when the ^55^Fe source is placed in direct contact with the APD and the bias voltage $V_{bias}$ is changed from 260 V to 410 V. The effect of $V_{bias}$ on $G_{APD}$ is evident: for higher $V_{bias}$ values, the histogram peaks center shift at higher MCA channels, meaning that in the input to the MCA, the voltage peaks arrive with a larger amplitude, i.e., $G_{APD}$ is increased. The height of the histogram peaks depends on the integration time, number of events, and energy resolution.

The histograms of the peak heights are then fitted with the Gaussian function to extract the centroid $Ch_{pk}$ and standard deviation $\sigma_{pk}$. The fit is performed by trimming the experimental histogram to a width that maximizes the $\chi^{2}$ test value with a Gaussian distribution. Taking into account the Gaussian function, the energy resolution δE/E is obtained from $\sigma_{pk}$ and $Ch_{pk}$ as follows:

(4) $$\frac{\delta E}{E}=\frac{\mathrm{FWHM}}{Ch_{pk}}=2.35\frac{\sigma_{pk}}{Ch_{pk}}\;,$$

with the FWHM, Full Width Half Maximum, of the Gaussian curve. The standard uncertainties $u(Ch_{pk})$ and $u(\sigma_{pk})$ and the Pearson correlation index ρ are obtained by the covariance matrix given by the least-square fitting method. These are then propagated [37] to δE/E:

(5) $$u^{2}\left(\frac{\delta E}{E}\right)={\left(\frac{\delta E}{E}\right)}^{2}\left[{\left(\frac{u(Ch_{pk})}{Ch_{pk}}\right)}^{2}+{\left(\frac{u(\sigma_{pk})}{\sigma_{pk}}\right)}^{2}+2\rho \frac{u(\sigma_{pk})}{\sigma_{pk}}\frac{u(Ch_{pk})}{Ch_{pk}}\right]\;.$$

This procedure is performed for all the following measurements.

**Figure 8:**
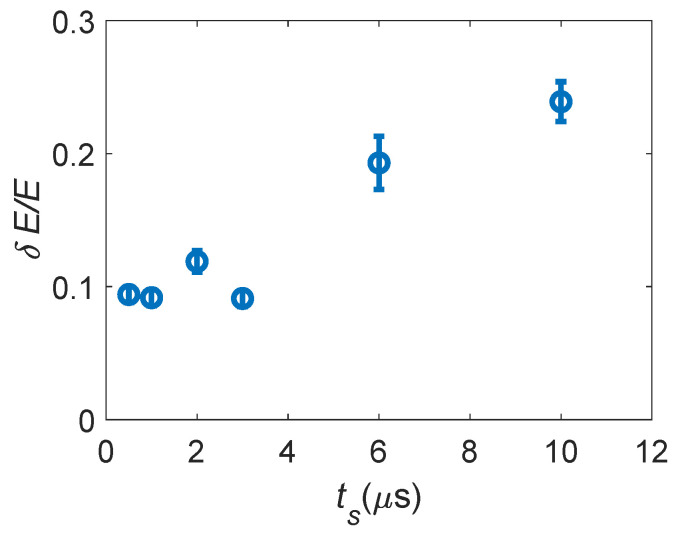
Energy resolution δE/E measured at room temperature by placing the ^55^Fe sample directly in contact with the APD, for different shaping times $t_{s}$.

The tuning of the Silena 7611 amplifier shaping time $t_{s}$ is then performed through δE/E. In Figure 8, δE/E is shown as a function of $t_{s}$ when the ^55^Fe is placed at room temperature in direct contact with the APD under test and it is biased with $V_{bias}=380$ V. The data in Figure 8 show that for higher $t_{s}$, δE/E increases, and thus the system characteristic time masks the real resolution of the APD. Since below $t_{s}\approx 3\;\mu$s, δE/E saturates, we conclude that for these $t_{s}$, the effect of the $t_{s}$ setting is negligible on δE/E. Thus, all the following measurements are performed setting the Silena 7611 gain at 210 and $t_{s}=1\;\mu$s.

Then, the system losses/gain must be properly calibrated in order to obtain $G_{APD}$. As discussed in Section 2, this would require the calibration of the gains and losses of each part of the system, but due to the difficulties and large uncertainties associated with this method, the use of a a reference APD is preferred. The APD used in this work as a reference is known to have $G_{APD,ref}=G_{APD}\left(382\right)=50\pm 1$ at 25 °C. In these conditions of *T* and $V_{bias}$, the MCA gives a peak centered at $Ch_{pk}=1051\pm 1$. Due to the linearity between the channel number and voltage of the MCA, this point can be used to obtain the calibration curve:

(6) $$G_{APD}=\frac{Ch_{MCA}G_{APD,ref}}{Ch_{ref}}=\frac{Ch_{MCA}\left(50\pm 1\right)}{1052\pm 1}\;.$$

**Figure 9:**
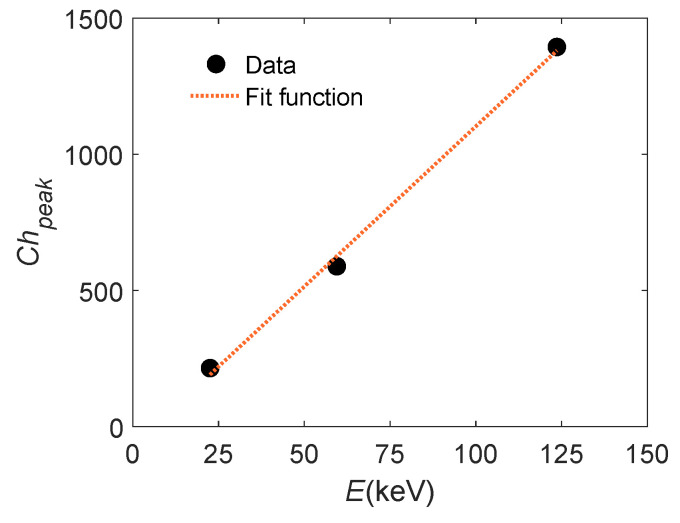
The position of the measured centroids $Ch_{pk}$ with respect to the energy *E* of the incident X-ray photons. Data are measured using the ^109^Cd, ^241^Am, ^57^Co samples with GAGG. Uncertainty bars are contained in the dimension of the symbols.

Finally, the final operative condition of the APD coupled with the GAGG crystal is preliminarily tested to verify the linearity of the system. The light yield by GAGG is in the optical spectrum, with a peak at ∼520 nm and intensity ∼50 photons/keV. This characteristic is exploited in the polarimeter configuration and can be used here to check the linearity of the MCA channel and of the system itself. The results are obtained by placing the GAGG crystal between the different radioisotope samples (listed in Table 1) and the APD. The ^55^Fe sample is not used in this case due to the lower energy of the emitted photons which is out of the X-ray band of interest. The result is shown in Figure 9. Data are fitted with the linear model $Ch_{pk}=mE$, with m=10.9(8) keV^−1^, where the number in parentheses is the numerical value of the standard uncertainty expressed in the unit of the quoted result. The good linear fit experimentally demonstrates the linearity of the whole system.

## 5. Results

The designed system is finally used to characterize the APD behavior in the whole *T*-$V_{bias}$ space of interest. In this section, the $G_{APD}$ measurements are shown and discussed. In particular, the experimentally obtained $G_{APD}\left(V_{bias},T\right)$ surface is used to obtain the parametric curve $V_{bias}\left(T\right)$ useful to stabilize $G_{APD}$ against the *T* variation in the operative conditions using a $G_{APD}$ control loop based on *T* measurements and $V_{bias}$ regulation.

**Figure 10:**
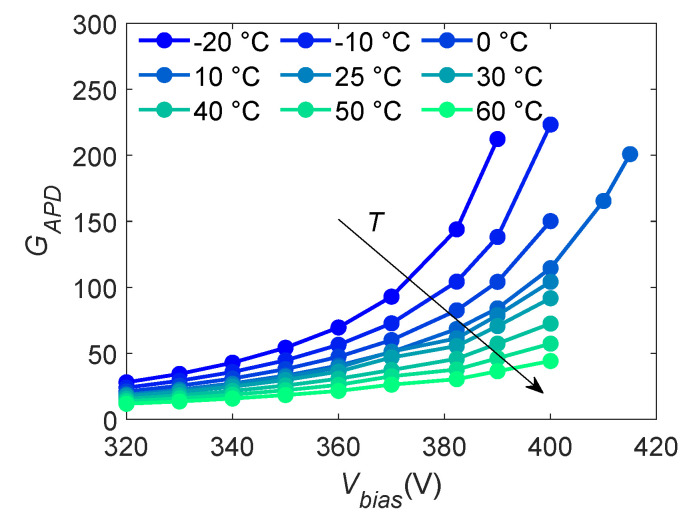
APD gain $G_{APD}$ measured for different biasing voltages $V_{bias}$ and temperatures *T*. Measurements are obtained by placing the ^55^Fe source in direct contact with the APD under test.

The APD gain $G_{APD}$ is measured as detailed in the previous Section, and the results are shown in Figure 10.

As expected from the theory, $G_{APD}$ increases when $V_{bias}$ is increased and *T* is decreased, whereas for what concerns the APD energy resolution measurements, the curves show a slight upward curvature with minima centered around $V_{bias}=380$ V. This is qualitatively expected due to the trade-off between the signal-to-noise ratio and noise dependence on $V_{bias}$.

**Figure 11:**
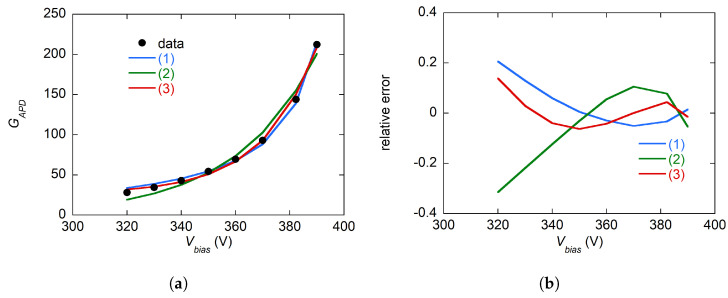
(**a**) Fit of the APD gain $G_{APD}$ measured at −20 °C with the following functions: (1) $G_{APD}=1/\left(1-{\left(V_{bias}/V_{0}\right)}^{n}\right)$ [29,30]; (2) $G_{APD}=a/\left(be^{\left(a\left(V0-V_{bias}\right)-1\right)}\right)$ [30]; (3) $G_{APD}=a+be^{\left(cV_{bias}\right)}$ [29]. (**b**) Relative error $\left(G_{fit}-G_{APD}\right)/G_{APD}$ obtained by the residuals of the fit shown in (**a**). The determination coefficients for the three fits are (1) $R_{1}^{2}=0.981$; (2) $R_{1}^{2}=0.995$; (3) $R_{1}^{2}=0.997$.

The dependence of $G_{APD}$ on $V_{bias}$ was fitted using different empirical models from the literature and with a standard exponential growth model $G_{APD}=a+be^{\left(cV_{bias}\right)}$ [29]. The results are shown in the −20 °C measurement in Figure 11. The residuals and determination coefficients obtained with the three models show that the standard exponential growth model is sufficient for the fit of the experimental data.

**Figure 12:**
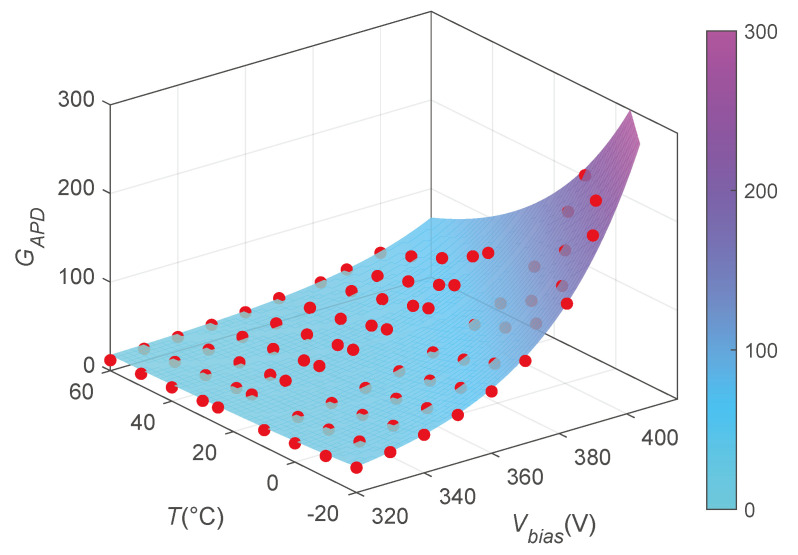
APD gain $G_{APD}$ measured for different biasing voltages $V_{bias}$ and temperatures *T*, placing the APD in direct contact with the ^55^Fe source. The experimental data are fitted with the function shown in Equation (2).

The same data points reported in Figure 10 are shown in the 3D plot in Figure 12 and fitted with the bi-dimensional function described by Equation (2).

The best fit is obtained with the Levenberg–Marquardt method: a=16.2, $b=6.0\times 10^{-5}$, $c=3.67\times 10^{-2}$ V^−1^, and $d=-2.65\times 10^{-2}$ °C^−1^. The parameter covariance matrix $U_{p}$ was determined through a Monte Carlo simulation [38] assuming the measured *T* and $V_{bias}$ as determination of stochastic variables with uniform distributions centered on the measured points and large ±1 °C for *T* and ±2 V and ±0.02% of the readings for $V_{bias}$. The width of the distribution is chosen accordingly with the experimentally measured maximum discrepancy between the AD590 sensors after the calibration process and the maximum error on the output voltage of the DT1471HET CAEN HV generator. In particular, for what concerns the distribution of the $V_{bias}$ points, the ±2 V is considered a strongly correlated systematic effect affecting all the points the same way, while the ±0.02% part is propagated as a random uncorrelated contribution. The simulation gives the following covariance matrix $U_{p}$:

(7) $$\left(\begin{array}{cccc} 0.04 & -1.54\times 10^{-6} & 6.44\times 10^{-5}\;V^{-1} & -3.09\times {10^{-5}}^{\circ }C^{-1}\\ -1.54\times 10^{-6} & 7.28\times 10^{-11} & -2.76\times 10^{-9}\;V^{-1} & 1.01\times {10^{-9}}^{\circ }C^{-1}\\ 6.44\times 10^{-5}\;V^{-1} & -2.76\times 10^{-9}\;V^{-1} & 1.16\times 10^{-7}\;V^{-2} & -4.30\times 10^{-8}\;{V^{-1}}^{\circ }C^{-1}\\ -3.09\times {10^{-5}}^{\circ }C^{-1} & 1.01\times {10^{-9}}^{\circ }C^{-1} & -4.30\times 10^{-8}\;{V^{-1}}^{\circ }C^{-1} & 4.62\times {10^{-8}}^{\circ }C^{-2} \end{array}\right)\;,$$

which is used for the evaluation of the uncertainties in the following data elaboration.

From the so-obtained $G_{APD}\left(T,V_{bias}\right)$, it is possible to determine the $V_{bias}$ control curve that can be used to keep $G_{APD}$ constant during typical *T* variations of orbiting CubeSats (from −20 °C to 60 °C [22,23]). This is obtained slicing the $G_{APD}\left(T,V_{bias}\right)$ surface at fixed $G_{APD}$ values:

(8) $$V_{bias}=\frac{1}{c}\left(\ln\left(G_{APD}-a\right)-\ln\left(b\right)-dT\right)\;.$$

**Figure 13:**
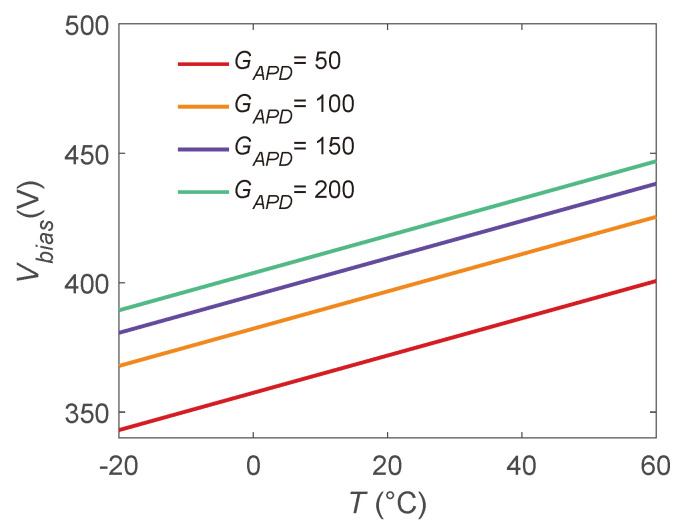
APD voltage bias $V_{bias}$ control curves for the stabilization of the gain $G_{APD}$ from −20 °C to 60 °C.

The so-obtained curves are shown for $G_{APD}=\left\{50;100;150;200\right\}$ in Figure 13. These are an example of the curves that can be obtained with the here-designed system. No uncertainties are evaluated on the control curves due to their application: define the $V_{bias}$ to apply to the APD when a certain *T* is measured. Their slope is α=−d/c, and thus APDs with large *c* (sensitivity to $V_{bias}$) and small |d| (sensitivity to *T*) are desired in order to reduce the span of the $V_{bias}$ values needed to stabilize $G_{APD}$.

However, the uncertainties in $G_{APD}\left(T,V_{bias}\right)$ are detrimental for the determination of $G_{APD}$ that can be obtained following one of the curves in Figure 13. To give an estimate of this detrimental effect, $V_{bias}\left(T\right)$ from Equation (8) is substituted in Equation (2) in order to evaluate the obtainable $G_{APD}$. Given the sensitivity coefficient vector:

(9) $$C_{p}=\left(\frac{\partial G_{APD}}{\partial a},\;\frac{\partial G_{APD}}{\partial b},\;\;\frac{\partial G_{APD}}{\partial c},\;\frac{\partial G_{APD}}{\partial d}\right)=\left(1,\;e^{\left(cV+dT\right)},\;bVe^{\left(cV+dT\right)},\;bTe^{\left(cV+dT\right)}\right)\;,$$

$u(G_{APD})$ can be evaluated as follows [39]:

(10) $$u\left(G_{APD}\right)={\left(C_{p}U_{p}C_{p}^{T}\right)}^{0.5}\;.$$

**Figure 14:**
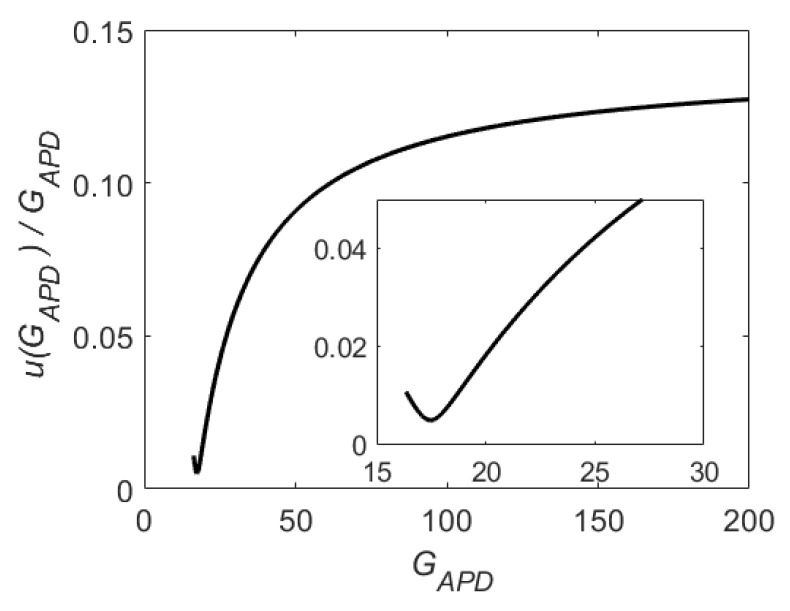
Relative standard uncertainty of the APD gain $u\left(G_{APD}\right)/G_{APD}$ obtainable with the stabilization curves reported in Figure 13. In the insert is a zoom of the curve near the minimum. For $G_{APD}\leq 16.2$, the curve is not defined; see Equation (8).

The temperature variation of $u\left(G_{APD}\right)/G_{APD}$ for fixed $G_{APD}$ values is well below the uncertainty of the evaluated uncertainty, and thus $u\left(G_{APD}\right)/G_{APD}$ here is assumed constant in temperature. Hence, the dependence of the relative standard uncertainty $u\left(G_{APD}\right)/G_{APD}$ on $G_{APD}$ is studied and the results are shown in Figure 14. A minimum is found in $u\left(G_{APD}\right)/G_{APD}$ at $G_{APD}=17$, and the uncertainty starts increasing rapidly as $G_{APD}$ is increased. A reduction in this uncertainty can be obtained with a more accurate measurement of $G_{APD}$, *T*, and $V_{bias}$, a denser *T* and $V_{bias}$ measurement grid, and the use of a $G_{APD}\left(T,V_{bias}\right)$ function that can better minimize $U_{p}$.

## 6. Conclusions

In this work, the issue of the calibration of the gain $G_{APD}$ and energy resolution δE/E of the APDs used in the readout of Compton scattering polarimeters for space applications has been addressed. In particular, since the use of these components for the CUSP mission exposes them to significant temperature, *T*, variations that affect their performance, the $G_{APD}$ dependence on *T* has to be experimentally determined. To address this, a system was developed to measure the $G_{APD}$ of the APDs over a wide range of temperatures (from −20 °C to 60 °C) and applied bias voltage $V_{bias}$ (from 260 V to 410 V) to investigate the possibility of stabilizing the gain by adjusting $V_{bias}$, thereby counteracting the fluctuations introduced by the *T* variations.

The designed and implemented system was tested in a climate chamber. The system consists of a charge amplifier to convert the charge signal into a voltage, a spectroscopy amplifier necessary to adjust the gain and appropriately shape the signal while minimizing pulse pileup effects, and an MCA to categorize the received pulses based on their amplitude.

The developed measurement system was used to characterize a test APD. The results show the $G_{APD}$ dependence, as a function of *T* and $V_{bias}$, that is qualitatively consistent with expectations for these devices. The obtained surface $G_{APD}\left(T,V_{bias}\right)$ was then fitted with a simple 2D exponential model to analyze its potential use for determining control functions useful for the stabilization of $G_{APD}$. The experimentally obtained curves were found to be simple linear functions; see Equation (8).

An uncertainty analysis, conducted using a combination of uncertainty propagation methods and distribution-based approaches, allowed the determination of the accuracy with which $G_{APD}$ can be stabilized with *T*. The results indicate that the uncertainties increase rapidly with the desired gain. However, it should be noted that this type of uncertainty primarily indicates a range of values within which $G_{APD}$ is expected to fall when polarized according to a chosen control curve. Given the high coefficient of determination obtained from the curve fitting, once a curve is followed, significant fluctuations in $G_{APD}$ with *T* are not expected due to the good correspondence between measured data and the fitting curves. Therefore, despite the seemingly large uncertainties, the stability of $G_{APD}$ will largely depend on the temperature measurement system of the APDs on the satellite and the stability and accuracy of the onboard high-voltage generator. For the CUSP mission, since measuring the number of incident photons is not necessary, achieving high accuracy in the APD gain is less critical than maintaining its stability. Uncontrolled variations in $G_{APD}$ would in fact be associated with erroneous photon detection at different energies. A thorough assessment of the control curves’ effectiveness will therefore be possible only after setting up the same system that will be used on the satellite to determine how the metrological characteristics of this would affect $G_{APD}$ stability.

## Figures and Tables

**Figure 2:**
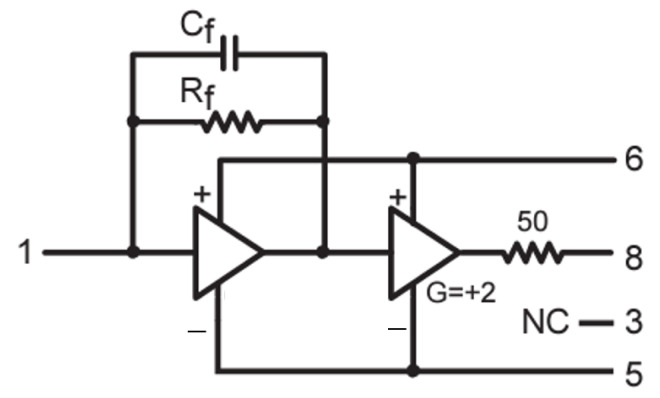
Charge preamplifier electrical circuit [34].

**Figure 3:**
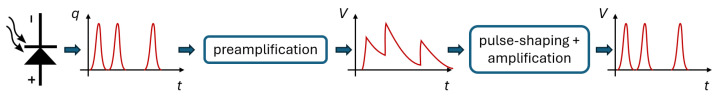
Block diagram of the signal preconditioning. The charge pulses in the output from the APD are passed to the preamplification stage to transform these into a voltage signal. Finally, this is passed to a pulse-shaping and voltage amplifier.

## Data Availability

The original contributions presented in this study are included in the article material. Further inquiries can be directed to the corresponding author.
